# Insurance Lemons for Physicians

**Published:** 1907-03

**Authors:** 


### Insurance Lemons for Physicians

IT has been brought to the attention of The Journal that one of the large insurance companies through its local agents has been made a party to a species of grand larceny in the effort to increase business among physicians. There has been reported one case of particular flagrancy which amounts to little less than ordinary theft on the part of the local agent and which if done in any other manner in everyday life would be susceptible of legal relief.

In brief, the method in this particular case was this: A physician was approached by the agent of a company which is ranked as "old line," and informed that a change was to be made in the corps of examiners; that he had been particularly decided upon for appointment, but that it would be necessary for him to take out a small policy. This was done and the application for appointment was made out, references given and all the requirements complied with. Within a week, it was stated to the physician, as on the authority of the general agent of the company, that the appointing power would visit Buffalo within a few days and that if an additional policy were immediately taken out to bring the whole insurance up to a certain amount, the appointing power would then have no objection to offer to immediate selection, and that then the work of examination of applicants for insurance in the company would be turned over to the physician. Further, it was outlined by the agent, after a conference with the general agent of the company, that examinations were to be made at a specified place at a specified rate; that the company he represented was one of the progressive organisations which had no part in the effort to reduce the examination fee to $3; that its fee was $5 and would remain so. The policies were taken out as suggested and the new application for appointment was made out, professional and business references being given as required.

Nothing more was heard of the matter for several weeks and when inquiry was made of the agent as to the status of the application, the physician was informed that the appointing power had been delayed in his arrival in Buffalo, and that it would be better to wait his arrival in order that a personal interview might the better clinch the appointment. From time to time excuses were made. After several months, during which time the physician merely waited with suspicion-sharpened patience, the original agent presented himself and placed the blame on the general agent of the company, stating that the delay was due to his neglect to bring the matter before the appointing power who had been in Buffalo. The physician expressed himself as of the belief that he had been swindled and the agent, after expressing regret that the general agent had acted in so unbecoming a manner, departed. The next move was the appearance, not wholly unexpected, of the general agent, who called to apologise for the statements made in the beginning by his agent, and saying that it was all a mistake; but that some day if a vacancy should occur, the physician would be considered for the position of examiner for the company.

This is a synopsis of the statement made to The Journal. Other matters were touched upon more in detail and they lead The Journal to believe that the securing of the premiums from the physician amounted to plain theft. There is every reason to believe that there are other physicians who have been buncoed in much the same manner and if so The Journal asks that the particulars be sent to this office. In the case in question, now that the agent and his stool pigeon have declared themselves, it is the intention to bring the entire matter before the officers of the company. Later, when additional facts are received in incontrovertible form, the company and the agents will be introduced to the profession. In the meantime it will be sufficient to say that the company in question is neither a New York nor a New Jersey corporation.

